# The evaluation of student-centredness of teaching and learning: a new mixed-methods approach

**DOI:** 10.5116/ijme.53cb.8f87

**Published:** 2014-08-14

**Authors:** Ana R. Lemos, John E. Sandars, Palmira Alves, Manuel J. Costa

**Affiliations:** 1 School of Health Sciences, University of Minho, Portugal; 2 Academic Unit of Medical Education, University of Sheffield, UK; 3 Institute of Education, University of Minho, Portugal

**Keywords:** Student-centred learning, espoused theories, theories-in-use, mixed methods

## Abstract

**Objectives:**

The aim of the study was to develop and consider the usefulness of a new mixed-methods approach to evaluate the student-centredness of teaching and learning on undergraduate medical courses. An essential paradigm for the evaluation was the coherence between how teachers conceptualise their practice (espoused theories) and their actual practice (theories-in-use).

**Methods:**

The context was a module within an integrated basic sciences course in an undergraduate medical degree programme. The programme had an explicit intention of providing a student-centred curriculum. A content analysis framework based on Weimer’s dimensions of student-centred teaching was used to analyze data collected from individual interviews with seven teachers to identify espoused theories and 34h of classroom observations and one student focus group to identify theories-in-use. The interviewees were identified by purposeful sampling. The findings from the three methods were triangulated to evaluate the student-centredness of teaching and learning on the course.

**Results:**

Different, but complementary, perspectives of the student-centredness of teaching and learning were identified by each method. The triangulation of the findings revealed coherence between the teachers’ espoused theories and theories-in-use.

**Conclusions:**

A mixed-methods approach that combined classroom observations with interviews from a purposeful sample of teachers and students offered a useful evaluation of the extent of student-centredness of teaching and learning of this basic science course. Our case study suggests that this new approach is applicable to other courses in medical education.

## Introduction

There is increasing emphasis on providing Higher Education that adopts a student-centred approach to teaching and learning. For example, the Bologna Process in Europe states “student-centred learning (SCL) is an approach to education, which aims at overcoming some of the problems inherent to more traditional forms of education by focusing on the learner and their needs, rather than being centred around the teacher’s input.”^1^ The importance of student-centredness for teaching and learning is also highlighted in several national and international recommendations for medical schools.^2^^-^^7^ For example, the General Medical Council in the United Kingdom recommends that learning should be “a process in which students are responsible for organising and managing their own learning activities and needs”^2^ The focus of SCL is on what and how the student is learning, with an expected outcome that there will be increased retention of the content and also that life-long learning will be developed by the student.^8^ In addition, there is improved student engagement and a shift in the balance of power in class, from teacher to learner.^9^Evaluating the student-centredness of teaching and learning is challenging since there is not a precise definition for “student-centredness.”^10^^-^^12^ However, Weimer provides a theoretical summary of the construct and offers five dimensions that can be useful for the evaluation of the student-centredness of teaching and learning:^8^ (a) the balance of power, with students involved in course decisions, including selection of content and assessment; (b) the function of content, with an emphasis on using content as a stimulus to learning and for the development of learning skills; (c) the role of the teacher, with a move towards the teacher becoming a learning facilitator that promotes student motivation and engagement, and creates an environment for learning; (d) the responsibility for learning, which should be placed upon students; and (e) the purpose and processes of evaluation, that should adopt the assessment for learning through a combination of both summative and formative assessment. Weimer’s dimensions to evaluate the student-centredness of teaching and learning have not previously been used in medical education and only a hybrid-version has been used in other contexts.^11^To achieve intended student-centred learning outcomes, teachers must conceptualise their teaching under a student-centredness perspective and teach accordingly.^13^ The theoretical views and beliefs of teachers about teaching (what they say that they would do in a certain situation), have been named “espoused theories”, whereas “theories-in-use” represent what they actually do.^13^^,^^14^ Evaluating whether the personal beliefs are expressed in actions requires assessing whether the theories-in-use correspond to the espoused theories.^14^ For example, teachers may hold firm beliefs that their focus is on facilitation of individual student learning, but teach through traditional lectures delivered to all students. This personal beliefs paradigm to understand the student-centredness of teaching and learning can be useful for staff development.^15^Studies in medical education which claim that a teaching or learning activity, including a whole course, is student-centred generally rely on information derived from student responses to questionnaires,^16^^-^^20^ or from a combination of semi-structured interviews and questionnaires.^21^ Some studies also infer the extent of student-centredness from differences in academic performance^19^^,^^20^or the relationship between the time students spent using a specific software and their final exam grades.^22^ However, these methods offer a limited view of the actual teaching and learning processes and there is a need for measures of student-centredness of teaching and learning beyond student evaluations.^23^ Studies in pre-university education have demonstrated the usefulness of alternative methods, such as classroom observations.^24^^,^^25^ Observing teachers in action and interviewing students and teachers are essential for the identification of the beliefs of teachers and how such beliefs are translated into practice. However, with the exception of a study comparing different instructional methods, ^26^ results from classroom observation methods are seldom reported in undergraduate medical education.

### Rationale for the study

We recognised the importance of student-centredness for teaching and learning but we had the challenge of how to evaluate this construct, especially from the paradigm of teacher espoused theories and theories-in-use. The aim of the study was to develop and consider the usefulness of a new mixed-methods approach to evaluate the student-centredness of teaching and learning. We underpinned our evaluation approach with Weimer’s dimensions of student-centredness and the paradigm of teachers’ espoused theories and theories-in-use^13^^,^^14^about facilitation of student-centred learning.^11^^,^^27^^,^^28^ For the context of our research, we chose a case study of a module within an integrated basic sciences course that had been consistently rated highly by students for being active in promoting student-centred learning.^29^ The course was part of a larger medical school programme with student-centred teaching and learning policies.^30^ For example, regarding classes, the policies define that “the learning activities should foster student interventions” through seminars or work in small groups.^30^

## Methods

### The case (context)

The case was a module on “muscle-skeleton” within the “Functional and Organic Systems I” (FOS I) course, a first year/second semester course of the undergraduate medical programme of the School of Health Sciences, University of Minho, in Portugal. FOS I was horizontally integrated at level nine in the integration ladder^31^ through an “organ systems-based” framework ^32^ to scaffold the learning of four major disciplinary areas: anatomy, physiology, biochemistry and histology.^29^ The course was sub-divided in three sequential blocks with similar length named modules.^29^ Teaching in a typical module followed a five step pedagogical cycle: i. overview tutorials to clarify learning objectives; ii. supervised or self-directed individual or group learning activities (including laboratory classes, group tutorials, literature readings, training of elementary clinical skills); iii. general disciplinary and multidisciplinary interactive lectures to identify any student difficulties related to understanding the content; iv. reflection and consolidation of learning; v. summative assessments. Patient vignettes were used extensively both in class activities – to trigger motivation and scaffold learning - and in assessment items in the summative assessments.^29^ The class observed in this study had a total of 167 students, of which 64.1% were females. The average age of the students was 18.7 years old.

### Data collection and analysis

Data was collected from individual interviews of teachers to identify their espoused theories, and classroom observations and a student focus group to identify the teachers’ theories in action. A content analysis framework based on Weimer’s five dimensions of student-centred teaching^8^ was used to analyse the data. ARL conducted the interviews and transcribed the interview audio-records verbatim. ARL and MJC categorized the materials using deductive analysis.^33^ARL and MJC independently read and coded the transcripts, discussing any discrepancies until a final consensus was agreed. Triangulation across the observation and interview data was conducted by ARL and MJC, discussing any discrepancies until a final consensus was agreed.Ethical approval was obtained from the University of Minho’s Ethics Subcommittee for health and life sciences: process SECVS - 021/2014. All teachers and students in the observed classroom sessions gave informed consent and all interview participants gave signed written consent. All participants were notified that the research would not identify participants by name.

#### (a) Interviews with teachers

A purposeful sampling method^33^^,^^34^ was used to identify teaching staff for interviews to ensure that there was a variety of teaching experience and that major disciplinary areas on the course were represented. We interviewed seven of the 36 (19%) course teachers from all the disciplinary areas. We targeted four novice teachers with three to four years of teaching experience and three experienced teachers with six to 11 years of teaching (four had presented papers in international education meetings, of whom one had educational publications in peer reviewed journals on approaches to facilitate student-centred learning).^35^ Teachers were interviewed after the conclusion of the course: two within two weeks and the others later, according to their availability.

#### (b) Classroom observations of teachers

The criteria used to identify classes for observations were coverage across all disciplinary areas, maximum sampling of course teachers, and diversity of classroom activities. Classes conducted by nine teachers, of whom seven were subsequently interviewed, were observed. The total time of observation was 34 hours, and included introductory tutorials (one hour in each disciplinary area), and classes within the steps ii and iii of the pedagogic cycle in the areas of anatomy (nine hours), histology (six hours), biochemistry (six hours), physiology (nine hours).The observer attended classes as a passive participant and used an open-ended protocol^36^ to annotate the strategies used by teachers within a framework derived from Weimer’s five dimensions of student-centred teaching. The observations were intended to document how the principles underlying student-centredness were put to use rather than to document the frequency of use of specific methods. All teachers gave verbal consent for the observations.

#### (c) Focus group of students

Student selection for the focus groups was conditioned by circumstances related to the academic calendar. Taking into considerations that the interview would take place at the end of the academic year and that we wished to maximise student participation, we initially sent an invitation to all students. However, after one reminder, we had only one reply. We then opted to approach students individually by email. We selected students who had taken the course for the first time and who had been active and critical participants in curricular discussions. We balanced for gender (two females) and included students from different secondary schools.

## Results

The student-centredness of teaching and learning on the course is presented, with supporting illustrative quotes, using the framework of Weimer’s five dimensions.

### The balance of power

In interviews, teachers mentioned the importance of engaging students in the learning process.

“We try to foster the students’ intellect, (…) force them to participate more in the class.” *(Teacher 1)*

“Because I think that [a presentation of a group assignment] worked well, the fact, for instance, I requested questions from students, and when students did not correspond, I then requested questions from the presenting group.” *(Teacher 2)*

“My concern [in classes] is to encourage the maximum participation of the student, i.e. that classes achieve the highest possible participation.” *(Teacher 3)*

As a means of transferring some control of the learning process to the students, teachers welcomed and valued the class as a place for discussion. There was a common perception of shared ownership of the class.

“I like the fact that (...) the issue does not get exhausted in that class, they can ask questions and I even do not know how to answer the questions, but then be able to individually, or even go with them and study the question that was put to me so we can find some response.” *(Teacher 6)*

"The system isn’t based on teacher. The system is based on the student.” *(Teacher 2)*

Classroom observations identified that students were frequently given autonomy in class, and teachers were available to answer questions. For example, in laboratory classes (histology and anatomy) instead of being told where to go and how much time to spend with materials that had been pre-selected by their teachers, the students could choose independently the sequence and the amount of time invested in the materials. Students in the focus group stated that they recognized that the classes were conducted in ways that required them to learn by themselves. For example, students considered oral presentation assignments as an important learning moment:

“As we explain things to other people we are forced to learn things much better than if we just had to listen to the content and then answer pre-defined questions.” *(Student A)*

Students also noted that there was a change in power relationships between teachers and students.

“These classes are very much ours.” *(Student B)*

The least student-centered aspects were the selection of course objectives and the design of the summative assessment program, which were entirely under teacher control, with teachers defining the timing and the amount of assessments.

“Mainly the teachers [take part on the design of the assessment program]”. *(Teacher 5)*

### The function of content

Teachers stated in the interviews that they used content to capture student curiosity and enhance student motivation Teachers were also concerned about pitching the level of difficulty of their questions so as not to make the class too difficult for the students.

“Make it [the subject] more interesting or make it a greater challenge to students.” *(Teacher 1)*

“We have to think carefully how to make their lives just a little more difficult.” *(Teacher 1)*

“(Ask) simple very general questions and the goal is that students will begin to go to places where they will have the content then start studying … until they gain interest and curiosity on the issues triggered by the questions.” *(Teacher 4)*

The biochemistry teachers considered that content should influence the development of student skills. The participation of students in class was seen as essential for student development, instead of only a way to assimilate content. The class activities of biochemistry included searching the literature to identify connections between molecules and disease, reading and discussing scientific papers and delivering oral presentations.

“Information that they gather at the moment, from their questions (…) and from the fact that they were thinking, it’s crucial.” *(Teacher 2)*

For example, in anatomy classes, as students circulated through materials, such as NMR scans and X-rays, they were constantly questioned about the underlying anatomical content and related clinical correlations. In the interviews, students referred to how teaching was often more directed to the development of skills instead of being centred on the scientific content.

“The aim of the activity is to prepare us to read scientific papers that will be our source of knowledge in the future.” *(Student B)*

“We learn to interpret.” *(Student A)*

### The role of the teacher

Teachers referred to themselves as learning facilitators and student guides in their interviews. One teacher explained that teachers should orientate students, but should not permanently shadow the student and prevent the student from learning how to take responsibility for their own learning.

“Teacher has responsibility on student learning, and then he should help them.” *(Teacher 1)*

(A teacher is someone who) “Guides [students] … and then it is up to the students to walk the path.” *(Teacher 2)*

Returning to the example of the histology and anatomy laboratories, observations revealed that there were always teachers in the vicinity to facilitate the students to explore the different materials. Students stated that they were comfortable with the design of classes, and they alternated peer-to-peer discussions with targeted questions to their teachers.

“I think that teacher is there with the orientation role (…) they [teachers] are guide you to the content that you will read.” *(Student B)*

“The teacher had an important role as give us the material, guide us through the subjects.” *(Student B)*

The responsibility for learning

Teachers stated that they gave students high responsibility in classroom activities.

“Students should take advantage and pose questions at that moment.” *(Teacher 2)*

“the goal is simply to put the student in contact with the images that will appear in the module or the nomenclature that will arise in the module, i.e. the student will do it by him/herself because we believe that from a cognitive point of view this is much richer if it is done by the student.” *(Teacher 5)*

Teachers attributed learning achievements to the effort and commitment of the students much more than to their personal commitment in teaching.

“Most of students’ work and learning didn’t result only by the work that was done with the teacher. Clearly, it is largely merit of the student who studies.” *(Teacher 2)*

The increased responsibility for learning was understood by the students as an opportunity to increase their knowledge.

“The reflections must be generated by us [students] and we are always posing questions.” *(Student A)*

“With our questions we [students] could achieve greater learning (…) in fact our role is paramount for the study.” *(Student B)*

Students agreed that the course demanded “responsibility of learning” and that the teaching approaches made them prepare for class.

“We need to arrive in class prepared. This really forces us to learn.” *(Student A)*

“[Teachers] posed questions and we should read the content at home.” *(Student C)*

### The purpose and processes of evaluation

According to teachers, summative assessments were used for grading but also to support students in identifying their learning gaps and to inform teaching. In comments related to the purpose and processes of evaluation, teachers described that evaluations should be used as a means to promote learning, especially formative assessment. Assessments were viewed by the teachers as diagnostic opportunities that were provided to the students, often through student peer-to-peer interactions.

“We have questions that specifically require them to discuss and interact.” *(Teacher1)*

“There are classes designed so they (the students) can ask questions and in those moments, they can understand what they know and what the others know.” *(Teacher 7)*

Teachers referred to assessments as a means to gauge that student learning was taking place.

“Assessment has something else that is more powerful but rarely seen in place, which is that assessment should also be like a learning moment, and that is not easy.” *(Teacher 2)*

“I conduct a type of Assessment which motivates students and let’s students know what is important for them to learn.” *(Teacher 3)*

However, one teacher (Teacher 4) was in dissonance with the others, emphasizing that the purpose of assessments was to rank students.

“The purpose of assessment is to… rank students.” *(Teacher 4)*

Students commented that classes were helpful for self-assessment of their strengths and weakness.

“[the activity] allowed me to see what I didn’t know so well, what I need to study more (…) and presented questions which help us to study better (…) [the activity] was important in order to prepare us to the exam.” *(Student A)*

Students reported that teachers provided instant feedback that worked as an important regulator for their learning.

“If students do not answer their questions, they [teachers] will say: «you’re doing bad in this part (…) you should study harder» (…). Sometimes teachers make questions and we answer right or wrong (…) teachers say: «you are well or not».” *(Student B)*

“Teachers will say: «you really need to study».” *(Student C)*

The congruence between the teachers’ espoused and theories-in-use is presented in Table 1, with Illustrative quotes.

**Table 1:** Congruence between the teachers’ espoused and theories-in-use according Weimer’s five dimensions

Dimension	Espoused theory	Theories-in-use
The balance of power	“[In classes] I give you something you give me something back and we not always have to agree” (Teacher 2).	Teachers invited student questions and stated that questioning was an important responsibility shared between faculty and students.
The function of content	“Ask questions which do not have to be complicated, but have to make the students to reason a bit” (Teacher 4).	Classroom observations revealed that teachers asked open questions that required students to evoke prior learning.
The role of the teacher	“Is more the role of a facilitator… to encourage students to go looking for things (...). Has the role (...) which is almost like a pointer in the sense that tells them where they should go and look for things and which things they should go and look for” (Teacher 3).	Teachers created opportunities for student peer-to-peer discussions but did not leave the students struggling by themselves.
The responsibility for learning	“[the method adopted in FOS I]is a method that gives them a plenty of freedom on the one hand, but also gives them a lot of responsibility on the other, because they cannot flee to much from the track in the time they have" (Teacher 5).	Students mostly attended classes with the content already studied. One example was a biochemistry class in which students were expected to read a scientific paper; the teachers were only present to orientate the activity and to clarify any questions from the students.
The purpose and processes of evaluation	“assessment (…) allows us, teachers, to understand to what extent we are passing on the information (…) it’s a moment of assessment (...) of the quality of our teaching, of the quality of our students, whether they are learning or not” (Teacher 1)	Classroom observation identified that teachers provided constant informal oral feedback in every class.

## Discussion

We conducted a case study as a proof of concept that a mixed-methods approach would be useful for the evaluation of student-centred teaching and learning in undergraduate medical education. The triangulation of our findings from teacher interviews (to identify their espoused theories) and classroom observations and a student focus group (to identify theories in action) revealed that the teachers’ vision of student-centredness and their actual teaching was coherent across Weimer’s theoretical model of five dimensions of student-centred teaching: “balance of power”, “the function of content”, “the role of the teacher”, “the responsibility for learning” and the “purpose and process of evaluation”.Teachers were aware of the importance of planning classes to engage and motivate students and of passing responsibility on to students. They did not consider themselves as mere content providers. Content was seen as a tool to develop student cognition, to learn general scientific skills (such as literature searches or reading and discussing scientific papers, preparing and presenting a work) and, very importantly, to facilitate the integration of disciplinary content by students. Assessments were considered important to steer student engagement in the learning process. The class observations showed that teachers did not conduct classes in prescriptive ways, instead classes provided opportunities for self-directed learning and peer-to-peer interactions. Teachers guided and stimulated the students, who were the focus of attention. The creation of an informal class environment stimulated students to engage in discussions about content, thus balancing the power in class. There was significant in-class time allocated for such interactions, in which teachers did not present materials, thus passing “responsibility for learning” to students. Frequently, teachers asked questions and provided formative feedback. In summary, there was a shared vision of the overall ethos of the medical programme by the teachers and this was translated into practice.The perceptions revealed by student interviews were also aligned with the above findings. For example, the students explicitly referred to teachers as their “guides” or “facilitators” and talked about their responsibility to prepare for class and develop their learning they were expected to. In terms of “the purpose and processes of evaluation” students confirmed that teachers provided constant feedback what was an opportunity for regulate their learning.There was one aspect in which there was dissonance with Weimer’s dimensions found when interviewing the students. Students considered they had little control over the selection of content, course policies and assessment methodologies. Nevertheless, students did not make comments that they needed to have such control, suggesting that they were satisfied with the current modus operandi of the course. This is reflected in the very positive results of the final year course questionnaires.^29^The comparison of findings across teacher interviews and class observations revealed there were common and personal beliefs and practices about student-centredness of teaching and learning. An example of a common belief identified in all of the interviewed teachers was the importance of the teachers’ role on the learning process. Teachers wanted to enhance student motivation and participation in their classes, and act as facilitators of the learning process. Interviewed students considered that all faculty shared an identical teaching philosophy aligned with student-centred principles. Such a shared vision suggests there is a common culture about teaching among the course faculty, despite the fact that this was a diverse faculty, which included both clinicians and academics. The faculty did not agree completely on the purpose of assessments. Whereas most teachers mentioned assessment as a tool to improve student learning, there was one faculty member who considered that assessment was only to classify the students. The fact that the study was able to capture diversity across faculty members suggests that the application of our mixed-methods approach can be useful for teacher development purposes.We consider that the main strength of this study is the complementary mixed-methods approach that evaluated both the of the student–centredness of teaching and learning on the course and also how teachers conceptualise their practice (espoused theories) and their actual practice (theories-in-use). This study’s research design uncovered relevant dimensions of teachers’ conceptualisations on the construct “student-centredness” which would not have been adequately identified in a questionnaire study. Given the time and resource investment required by this new methodology, we found it a feasible and useful approach to evaluate the student-centredness of teaching and learning on a course within the scope of this case study. As a practice point, we suggest that it may be of use to other courses in other institutions of medical education. In addition, the results of the observation of teachers would be available to be used for the benefit of developing the teachers, as a means to provide formative feedback about their teaching. We did not explore this possibility in the current study.We recommend that further research is conducted in more courses and institutions to identify if the application of this approach can shed new light into our understanding of how teaching and learning is delivered in courses that describe themselves as student-centred, as well as identifying the extent to which the espoused theories of teachers are coherent with their theories-in-use.We are aware that our study has several limitations. Interviews with more students and teachers and repetition of interviews to ensure saturation would provide more validity and reliability to our findings. Indeed, a single focus group with four students is probably insufficient to represent the population or to reach data saturation, but we had difficulties with student availability, as students leave for summer holidays shortly after the end of the course. An important key limitation is that we did not evaluate outcome indicators of the course’s student-centredness. However, the results of student ratings over the last ten years has shown a consistent high level of student satisfaction with their teaching.^29^

## Conclusion

There was a shared and coherent vision on student centredness between the course and programme policies, the beliefs of the teachers, classroom practice and student perceptions. The different pieces of information collected through complementary methods strengthen the argument that the course can be described as student-centred.Our aim was to develop and consider a new approach to evaluate the student-centredness of teaching and learning in undergraduate medical courses. We consider that the mixed-methods approach that we have developed is potentially useful as an evaluation tool, especially to identify the espoused theories of teachers, both individually and collectively as a group, and the theories in action. The combination of teacher and student interviews with class observations may also prove to be a feasible complementary approach to current course evaluations of student-centredness of teaching and learning based on questionnaires. Despite the fact that this is the first case study conducted to evaluate a new approach, we have gathered information that provides a richer account on the diversity of the student-centredness of teaching and learning on the course and this information can be fed back to the teaching faculty and course directors, for purposes of course development.

## 

### Conflict of Interest

The authors declare that they have no conflict of interest.
